# Factors Associated with Maternal Serum Levels of Perfluoroalkyl Substances and Organochlorines: A Descriptive Study of Parous Women in Norway and Sweden

**DOI:** 10.1371/journal.pone.0166127

**Published:** 2016-11-08

**Authors:** Hilde B. Lauritzen, Tricia L. Larose, Torbjørn Øien, Jon Ø. Odland, Margot van de Bor, Geir W. Jacobsen, Torkjel M. Sandanger

**Affiliations:** 1 Department of Public Health and General Practice, Norwegian University for Science and Technology, Trondheim, Norway; 2 Department of Community Medicine, University of Tromsø – The Arctic University of Norway, Tromsø, Norway; 3 Section of Health and Life Sciences, Vrije Universiteit, Amsterdam, The Netherlands; 4 School of Health Systems and Public Health, University of Pretoria, Pretoria, South Africa; 5 NILU-Norwegian Institute for Air Research, Tromsø, Norway; Gentofte Hospital, DENMARK

## Abstract

**Introduction:**

Perfluoroalkyl substances (PFASs) and organochlorines (OCs) are ubiquitous and persistent in the environment and proposed endocrine disrupting chemicals (EDCs). They can be transferred across the placenta during pregnancy, and studies suggest that the prenatal period may be particularly sensitive for influences on fetal growth and development. Several studies have investigated socio-demographic and pregnancy related factors associated with maternal serum PFAS and OC levels, but few studies have been conducted in time periods with increasing emissions of PFASs and recent emissions of OCs.

**Methods:**

Serum from 424 pregnant women participating in the NICHD Scandinavian Successive Small-for-gestational Age (SGA) births study was collected in 1986–1988, and analyses of two PFASs and six OCs were conducted. Associations between EDCs and geographic, time dependent, socio-demographic and pregnancy related variables were evaluated by using multivariable linear regression models.

**Results:**

Previous breastfeeding duration, time since last breastfeeding period, sampling date and country of residence were important factors associated with serum levels of PFOS and PFOA. Smoking status and pre-pregnancy BMI were negatively associated with PFOS, and maternal height was borderline negatively associated with PFOS and PFOA. Glomerular filtration rate (GFR) was negatively associated with PFOS in a sub-sample. Maternal serum levels of OCs were positively associated with maternal age, and negatively associated with previous breastfeeding duration and sampling date. Smoking had a consistently negative association with PCB 118 in a dose-dependent manner. Education level, pre-pregnancy BMI and alcohol consumption varied in importance according to the compound under study.

**Conclusions:**

Several maternal factors, including potentially modifiable factors, markers of pregnancy physiology and factors also related to perinatal outcomes were associated with EDC levels. Results from this study are relevant to populations with still high PFAS and OC levels, i.e. developing countries. Moreover, we can use this knowledge about associated factors on emerging EDCs with similar properties.

## Introduction

Perfluoroalkyl substances (PFASs) are synthetic chemicals that have been widely used in consumer products including lubricants, paper products and textiles since the 1950s [1]. Numerous PFASs are persistent substances, with approximate human half-lives of 2–5 years [2]. Organochlorines (OCs) comprise several substances, including polychlorinated biphenyls (PCBs) and pesticides. PCBs have been produced for commercial use in paints, plastics and electrical transformer fluids, whereas pesticides were used to control pests and disease [3]. PFASs are proteinophilic, primarily bound to albumin in serum and reside in blood, liver and kidneys [4], whereas OCs are lipid soluble and accumulate in fatty tissues [5]. PFASs and OCs can cross the placental barrier [6, 7], and *in utero* exposure have been associated with adverse effects of growth and development in both animal and epidemiological studies [8–10]. These environmental pollutants have been categorized as endocrine disruptive chemicals (EDCs) due to their disruption in the regulation of estrogen and thyroid hormones [11, 12]. In 2012, a report from the United Nations Environment Programme (UNEP) and the World Health Organization (WHO) identified EDCs as an emerging health challenge to vulnerable individuals in society, particularly fetuses and children [13]. Maternal serum EDC levels are relevant biomarkers of fetal exposure.

There are large inter-individual differences in maternal serum levels of EDCs, but reasons behind the large variation have not been consistent. A better understanding of factors associated with serum levels of EDCs would improve the basis for advices with the purpose to lower the body burdens of young women during pregnancy. This knowledge may also provide information regarding exposure routes in addition to distribution and elimination of EDCs in the body.

Most previous studies on factors associated with PFAS and OC levels were conducted in post-ban periods when contaminant levels were declining in most Western countries. Important sources of exposures and associated maternal factors will change over time as emission history is changing. New knowledge about associated factors in historical periods of increasing PFAS emissions and recent OC emissions is important because biomonitoring studies have revealed minimal temporal declines and even increases in PFAS and OC levels among some populations, i.e. some developing countries [3, 14]. Moreover, we can use this knowledge about associated factors on new emerging EDCs with similar properties. Although epidemiologic studies have reported associations between maternal serum levels of PFASs and OCs and indices of fetal growth [15, 16], potential confounders of these associations are not consistently reported and important to examine. This descriptive study of Scandinavian parous women examines factors associated with maternal serum levels of PFASs and OCs from years of continued emissions of PFASs and recent emissions of OCs (1986–1988) [17].

## Materials and Methods

### Ethics statement

All participants provided written informed consent for continued use of data and biomaterial, which was documented at the first study visit. The study, including the consent procedure, has been reviewed and approved by the Central Norway Regional Committee for Medical and Health Sciences Research Ethics (REK Midt 2010/1449-5).

### Study area and population

**Fig 1:**
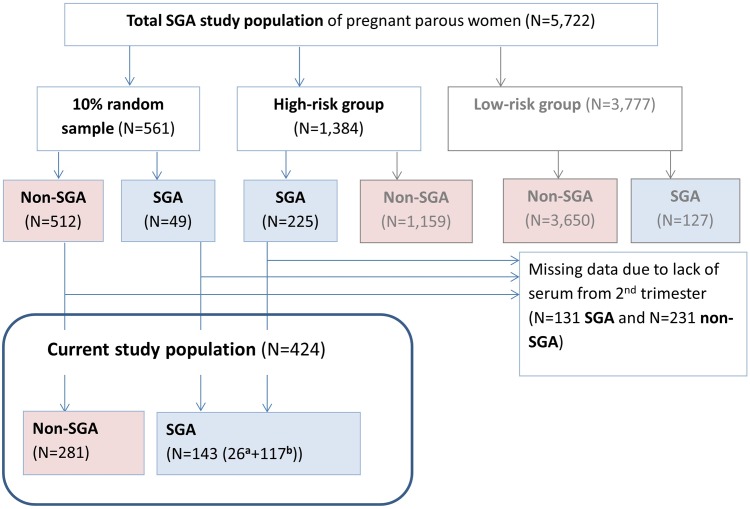
Flow chart of the participant selection for PFAS and OC analysis. ^1^From 10% random sample.^2^From High-risk group.

Participants were from the NICHD Scandinavian Successive Small- for- Gestational Age (SGA) births study; a population based prospective multicenter study conducted in Trondheim and Bergen (Norway) and Uppsala (Sweden) (1986–1988) [17]. The SGA births study was designed to study the etiology and consequences of intrauterine growth restriction. Eligible participants included para 1 and para 2 women of Caucasian origin who spoke one of the Scandinavian languages and had a singleton pregnancy. In total, 5,722 women were eligible and made the first appointment, from which three groups were defined: a 10% random sample (n = 561); a group at high risk for SGA birth (n = 1,384), and a rest of population group (n = 3,777). Both the random sample and high risk groups were included for detailed follow-up. The high risk for SGA birth group was defined by one or more of the following risk factors: a) a prior low birth weight (LBW) child, b) maternal cigarette smoking at conception, c) low pre-pregnancy weight (<50 kg), d) a previous perinatal death, or 5) the presence of chronic maternal disease. Serum samples from n = 424 mothers were randomly selected for PFAS and OC analyses including a 2:1 ratio of non-SGA births to SGA births for future analysis (Fig 1).

### Data on maternal characteristics

Data on socio-demographics, body mass index (BMI), lifestyle variables, and previous obstetric history were collected at first study visit. Socio-demographic variables included maternal age (continuous; years) and level of education (ordinal; 5 levels from low to high). Body weight and height were reported and pre-pregnancy BMI was calculated as weight in kilograms divided by height in meters squared (continuous; kg/m^2^). Lifestyle variables included smoking status at conception (categorical; smokers or non-smokers), number of cigarettes smoked at conception and in 2^nd^ trimester, and alcohol consumption during pregnancy (ordinal; 5 levels from low to high). Smokers were women who at first study visit reported daily smoking at the time of conception. Obstetric variables included parity (binary; 1 or 2), previous breastfeeding duration (continuous; months) and time since end of last breastfeeding period (continuous; per year). We categorized serum collection by sampling date (continuous; for each 100 days since study inception) and gestational age at serum sampling (completed weeks).

### Measurement of PFAS and OCs

Maternal serum samples were collected during the 2^nd^ trimester at approximately week 17 (Table 1), and stored at -80°C for later analysis. In total, a random selection of n = 424 serum samples were included for PFAS and OC analyses (Fig 1).

**Table 1:** Maternal characteristics of the current sample of Scandinavian parous pregnant women in the SGA-study (N = 424).

		% or mean (range)
**Gestational age**^1^ **at serum sampling**		17 (13–20)
**Sampling date**	1986	41.2%
	1987	49.8%
	1988	7.8%
**Country of residence**	Norway	62.5%
	Sweden	37.5%
**Maternal age (years)**		29.0 (18–41)
**Maternal height (cm)**		166 (150–182)
**Maternal pre-pregnancy BMI (kg/m**^**2**^**)**		21.5 (16–33)
**Education level**	<9 years	16.5%
	9+1–2 years	28.3%
	9+3 years	21.2%
	Higher education, non-university level	24.8%
	Higher education, university level	8.0%
**Parity**	1	68.9%
	2	31.1%
**Smoking at conception**	No	54.5%
	Yes	45.5%
Number of cigarettes per day at conception		5.4 (0–25)
**Smoking in early 2**^**nd**^ **trimester**	No	58.7%
	Yes	40.1%
Number of cigarettes per day at 2^nd^ trimester		4.1 (0–25)
**Alcohol consumption during pregnancy**^2^	Never	47.4%
	< once a month	33.7%
	About once every month	9.2%
	2–3 times per month	4.7%
	≥ once a week	1.9%
**Previous breastfeeding duration (months)**		7.4 (0–24)
**Time since end of last breastfeeding period**	<2 years	42.7%
	2–4 years	28.3%
	>4 years	21.9%

^1^Gestational age based on ultrasound measurements (completed weeks)

^2^Infomation collected in week 33 of pregnancy

#### Chemical analyses of PFAS

Analyses were performed at the laboratories of Norwegian Institute for Air Research, Tromsø, Norway (NILU). PFASs measured were perfluorooctanoate (PFOA) and perfluorooctane sulfonate (PFOS). Samples were analyzed using sonication-facilitated liquid-liquid extraction, activated ENVI-carb clean-up [18], quantified and analyzed by ultrahigh pressure liquid chromatography triple-quadrupole mass-spectrometry (UHPLC-MS/MS).

Sample preparation and extraction were performed as described by Hanssen et. al [19] except for minor volume changes.

The quantification was conducted with the LC Quan software, version 2.6.0 (Thermo Fisher Scientific Inc, Waltham, MA, USA). The internal-standard addition method with isotope-labeled PFASs was used to quantify the contaminants [19]. Concentrations of PFASs in all samples were within the linear range of the instrument and the calibration curve. In the mass spectrometry analyses, a second mass transition served to confirm compound specificity for each compound. The quality of the analysis was verified by repetitive analysis of blank samples and reference samples (SRM 1957, NIS, Gaithersburg, MD, USA). Participation in the AMAP ring test program and results from SRM material indicates a coefficient of variation (CV) of 15% for PFOA and 10% for PFOS. The linear PFOS isomers were chromatographically separated from the branched isomers and quantified separately. Unless otherwise specified, the PFOS results discussed are the sum of linear and branched isomers.

#### Chemical analyses of OCs

OCs were analyzed using the POPs method E-446 (ISO 1 7025 accreditation) at the Institut National de Santé Publique du Quebec, Centre Toxicologie, Quebec. This laboratory is the organizer of the AMAP ring program. OCs measured were hexachlorobenzene (HCB), oxychlordane, PCB 52, PCB 101, PCB 118, PCB 153, PCB 156, PCB 170, PCB 180, *p*,*p’*-dichlorodiphenyldichloroehylene (*p*,*p’*-DDE), *p*,*p’-*dichlorophenyltrichloroethane (*p*,*p’*-DDT), *beta*-hexachlorohexane (*β*-HCH) and *trans*-nonachlor (*t-*NC). Only OCs detected in more than 99% of the samples and used in the regression analyses were included in Table 1. Because of high correlation between PCBs we chose to use and report PCB 118, representing dioxin-like PCBs and PCB 153, representing non-dioxin like PCBs. In short, 0.5–1 ml serum sample was extracted using hexane (2x6 ml), ethanol (2 ml) and saturated ammonium sulphate solution (2 ml). This method is a slight modification of the one described by Sandanger et al. [20], where the samples were cleaned up using 1 g of activated fluorisil on an automated Liquid handler system before GC-MS analysis described by Sandanger et al. [21]. Results from the AMAP ring test program and SRM material indicate a CV of 5–10% for the OCs analyzed.

### Statistical analyses

The distribution of PFAS and OC levels closely followed a log-normal distribution. We therefore transformed data accordingly. We used wet weight concentrations of PFASs and lipid-adjusted serum concentrations of OCs [22]. As a sensitivity analysis we used wet weight values of OCs and adjusted for total lipids as an independent variable, but neither point estimates nor 95% confidence intervals (95% CIs) showed considerable change (data not shown).

Total lipid values were calculated based on measurements of triglycerides and cholesterol: total lipids = 1.33*triglycerides + 1.12*cholesterol + 1.48 (g/l) [22]. This formula showed good correlation with complete formulas including phospholipids [23].

We used multivariable linear regression to estimate associations between maternal characteristics and serum PFAS (PFOA and PFOS) and OC (PCB 118, PCB 153, *p*,*p’*-DDE, *t*-NC, HCB and *β-*HCH) levels, and reported adjusted estimates and 95% confidence intervals (CIs). Based on a priori knowledge, the following co-variates were included in adjusted analyses: serum sampling date, study site (Norway/Sweden), maternal age, education level, maternal height and pre-pregnancy body mass index (BMI), smoking status at conception, alcohol consumption during pregnancy, parity (1 vs. 2), previous breastfeeding duration and time since end of last breastfeeding period. We evaluated linear model assumptions using diagnostic plots of the residuals and checked the covariates for multicollinearity by variance inflation factors (VIFs). We calculated percent change in PFAS and OC levels for each independent variable by exponentiating regression coefficients, subtracting 1 and multiplying by 100.

In sub-analyses we investigated potential dose-response relationships between smoking intensity (number of cigarettes smoked at conception and in early 2^nd^ trimester) and levels of PFOS, PCB 118 and *β*-HCH. As a sensitivity analysis we used serum cotinine levels measured from samples collected at first study visit available from n = 88 women to further examine the association between smoking and PFOS, PCB 118 and *β*-HCH levels.

As markers of pregnancy physiology we examined glomerular filtration rate (GFR) in n = 88 of the samples. We calculated GFR (mL/min per 1.73 m^2^) using the Cockroft-Gault (GFR-CG) formula (GFR-CG = (140-age) x weight (g) x 1.04/serum creatinine (μmol/L)).

We performed statistical analyses using SPSS statistical software, version 22 (IBM SPSS Inc. Chicago, IL, USA).

## Results

### Maternal baseline characteristics

Maternal baseline characteristics are presented in Table 1. The gestational age at serum sampling varied from 13 to 20 weeks, with a mean gestation age of 17 weeks. A larger proportion of participants came from Norway (63%), while the majority of participants were pregnant with their second child (69%). Nearly half of all participants reported smoking at conception (46%), with a slight reduction by first visit in early 2^nd^ trimester (40% smokers). In gestational week 33, more than half of all study participants reported at least some alcohol consumption (47% no alcohol).

### Serum PFAS and OC levels

Serum levels of two PFASs (PFOA and PFOS) and six OCs are presented in Table 2. All PFASs and OCs reported were detected in 100% of the samples, with the exception of *β-*HCH levels which were detected in 99% of the samples. In wet weight values, PFOS was the dominating compound, followed by PFOA, *p*,*p’*-DDE, PCB 153, *β*-HCH, HCB, PCB 118 and *t*-NC.

**Table 2:** Maternal serum levels of PFASs (ng/ml) and OCs (ng/ml and ng/g lipids) in the SGA study (N = 424).

	Wet weight (ng/ml)	Lipid weight (ng/g lipid)	LOD^1^	%>LOD^2^
	Median (range)			
**PFOA**	1.82 (0.31–7.97)	-	0.03	100
**PFOS**	12.3 (0.95–59.6)	-	0.03	100
**PCB 118**	0.08 (0.03–0.27)	13.9 (5.38–86.2)	0.01	100
**PCB 153**	0.52 (0.17–1.40)	89.9 (31.0–212)	0.01	100
***p*,*p'*-DDE**	1.30 (0.10–11.0)	223 (16.7–1791)	0.09	100
**HCB**	0.10 (0.04–0.38)	17.7 (6.98–73.0)	0.04	100
***β-*HCH**	0.13 (<LOD-0.76)	22.1 (<LOD-134)	0.01	99
***t*-NC**	0.04 (0.01–0.14)	6.51 (1.82–25.2)	0.01	100

^1^LOD: Limit of detection (ng/ml)

^2^%>LOD: percentage of samples in which the analyte was detected

### Maternal factors associated with PFAS and OC levels

#### PFASs

In adjusted analyses, Swedish women had 39% higher PFOA levels and 67% higher PFOS levels compared to their Norwegian peers. Previous breastfeeding duration was negatively associated, while time since last breastfeeding period was positively associated with PFOA and PFOS. An increase in both PFOA and PFOS per 100 days from study enrollment was observed (2.8% and 5.0% respectively). Smokers had on average 21% lower levels of PFOS than non-smokers (Table 3), and in sensitivity analyses, we found a linear relationship between increasing number of cigarettes smoked at conception (p = 0.015) and in early 2^nd^ trimester (p = 0.006) and decreasing PFOS levels (Table 4). However, this finding was not replicated in a sensitivity analyses of n = 88 women with available cotinine levels from 2^nd^ trimester (p = 0.113) (Table 4). Adjusted for maternal height, pre-pregnancy BMI and gestational age at serum sampling, GFR was negatively associated with PFOS in the same sub-sample (n = 88) (Table 5). Pre-pregnancy BMI was negatively associated with PFOS levels in multivariable linear regression model (Table 3).

**Table 3:** Adjusted^1^ associations from multivariable linear regression models for sociodemographic and pregnancy-related variables and ln-transformed maternal PFAS (ng/ml) and OC levels (ng/g lipids) in serum collected in 2nd trimester (n = 424).

	Ln (PFAS (ng/ml))		Ln (OC (ng/g lipid))					
	PFOA	PFOS	PCB118	PCB153	*p*,*p'*-DDE	HCB	*β*-HCH	*t*-NC
	% change (95% CI)	% change (95% CI)	% change (95% CI)	% change (95% CI)	% change (95% CI)	% change (95% CI)	% change (95% CI)	% change (95% CI)
Sample date (per 100 days)	**2.8 (0.7, 4.9)**	**5.0 (2.4, 7.7)**	-1.6 (-3.4, 0.2)	**-2.5 (-3.7, -1.3)**	**-7.2 (-9.7, -4.7)**	**-2.4 (-3.7, -1.0)**	**-4.4 (-5.9, -2.8)**	-0.5 (-2.2, 1.3)
Country of residence								
Norway	*ref*.	*ref*.	*ref*.	*ref*.	*ref*.	*ref*.	*ref*.	*ref*.
Sweden	**39 (27, 53)**	**67 (49, 88)**	8.0 (-0.7, 17)	**43 (35, 52)**	2.0 (-11, 16)	-2.5 (-8.7, 4.2)	7.4 (-0.5, 16)	**-9.4 (-16, -1.7)**
Maternal height (per 10 cm)	*-6*.*7 (-14*, *0*.*3)*	*-8*.*3 (-16*, *0*.*6)*	5.6 (-1.1, 13)	1.1 (-3.4, 5.9)	10 (-0.6, 22)	1.9 (-3.3, 7.3)	0.1 (-5.7, 6.2)	1.1 (-5.2, 7.7)
Maternal BMI (per kg/m^2^)	-1.4 (-3.0, 0.3)	**-2.3 (-4.2, -0.2)**	-0.5 (-1.9, 1.0)	**-2.6 (-3.6, -1.6)**	-1.1 (-3.3, 1.1)	-0.3 (-1.4, 0.9)	1.0 (-0.3, 2.4)	**-2.0 (-3.4, -0.6)**
Maternal age	-0.1 (-1.5, 1.3)	-0.1 (-1.9, 1.7)	**2.5 (1.2, 3.8)**	**2.9 (2.0, 3.8)**	**5.5 (3.5, 7.6)**	**2.6 (1.6, 3.7)**	**3.2 (2.0, 4.4)**	**3.5 (2.3, 4.8)**
Smoking at conception								
No	*ref*.	*ref*.	*ref*.	*ref*.	*ref*.	*ref*.	*ref*.	*ref*.
Yes	-4.7 (-15, 5.0)	**-21 (-37, -7.4)**	**-26 (-38, -16)**	0.9 (-5.3, 6.6)	3.0 (-11, 15)	-4.4 (-12, 2.5)	**9.2 (1.9, 16)**	7.0 (-1.1, 14)
**Alcohol consumption** (5 groups from low to high)^3^	0.6 (-4.2, 5.5)	-0.4 (-6.2, 5.8)	**5.1 (0.8, 9.7)**	3.5 (0.4, 6.6)	3.3 (-3.2, 10)	3.2 (-0.2, 6.8)	2.5 (-1.3, 6.6)	**5.6 (1.3, 10)**
**Education level** (5 groups from low to high)^2^	2.0 (-2.4, 6.6)	3.0 (-2.5, 8.7)	2.3 (-1.6, 6.3)	1.6 (-1.1, 4.4)	**7.0 (0.9, 14)**	1.4 (-1.7, 4.6)	3.3 (-0.3, 7.0)	**4.8 (0.9, 8.8)**
**Parity**								
1	*ref*.	*ref*.	*ref*.	*ref*.	*ref*.	*ref*.	*ref*.	*ref*.
2	-3.1 (-13, 7.9)	-6.7 (-19, 6.8)	8.1 (-1.8, 19)	7.2 (0.3, 15)	7.6 (-7.1, 25)	2.1 (-5.4, 10)	1.2 (-7.2, 10)	0.3 (-8.6, 10)
**Previous breastfeeding duration** (per month)	**-1.3 (-2.3, -0.2)**	**-1.6 (-2.8, -0.3)**	**-2.3 (-3.2, -1.4)**	**-2.1 (-2.7, -1.4)**	**-3.5 (-4.9, -2.2)**	**-1.9 (-2.6, -1.2)**	**-2.7 (-3.6, -1.9)**	**-1.0 (-1.9, -0.1)**
**Time since last breastfeeding period** (per year)	**4.8 (2.9, 6.8)**	**2.8 (0.5, 5.2)**	-0.5 (-2.1, 1.1)	-0.4 (-1.5, 0.7)	-0.6 (-3.0, 1.9)	-0.2 (-1.5, 1.0)	0.2 (-1.2, 1.7)	-0.01 (-1.6, 1.6)

^1^Multivariable models were adjusted for all variables included in the table.

^2^Education level (ordinal): 1 = <9 years, 2 = 9–11 years, 3 = 12 years, 4 = higher education, non-university level, 5 = higher education, university level.

^3^Alcohol consumption during pregnancy (ordinal): 0 = never, 1 = <once a month, 2 = once a month, 3 = 2–3 times a month, 4 = >once a week.

**Table 4:** Adjusted^1^ associations from multivariable linear regression models between smoking intensity and ln-transformed maternal PFOS, PCB 118 and β-HCH levels in serum collected in 2nd trimester (n = 424).

	PFOS		PCB 118		*β*-HCH	
	% change (95% CI)	p-value	% change (95% CI)	p-value	% change (95% CI)	p-value
**Smoking status at conception**						
**no smoking**	*ref*.		*ref*.		*ref*.	
**1–5 cig/day**	-11 (-29, 11)	0.292	-14 (-23, -4.9)	0.004	1.1 (-12, 16)	0.876
**6–10 cig/day**	-9.1 (-21, 4.1)	0.166	-20 (-25, -14)	<0.001	13 (4.3, 23)	0.003
**>10 cig/day**	-15 (-26, -2.7)	0.019	-24 (-29, -19)	<0.001	13 (3.7, 23)	0.005
***p for trend***		*0*.*015*		*<0*.*001*		*0*.*001*
**Smoking status at week 17**						
**no smoking**	*ref*.		*ref*.		*ref*.	
**1–5 cig/day**	-7.7 (-23, 11)	0.384	-21 (-28, -15)	<0.001	1.1 (-10, 13)	0.845
**6–10 cig/day**	-17 (-28, -4.5)	0.010	-23 (-28, -18)	<0.001	15 (5.9, 26)	0.001
**>10 cig/day**	-13 (-26, 1.9)	0.084	-25 (-31, -20)	<0.001	6.9 (-2.9, 18)	0.173
***p for trend***		*0*.*006*		*<0*.*001*		*0*.*010*
**Serum cotinine levels at 2^nd^ trimester**^2^	-2.0 (-4.5, 0.5)	0.113	-1.8 (-3.0, -0.6)	0.004	0.8 (-1.1, 2.7)	0.428

^1^Adjusted for maternal pre-pregnancy BMI, country of residence and maternal PCB 153-levels.

^2^Only analyzed for a subset of 88 women. Numbers shown are percent change in POPs levels for each 100-unit increase in serum cotinine (range 0–1856).

**Table 5:** Adjusted associations from multivariable regression models between GFR, maternal height and pre-pregnancy BMI, and ln-transformed maternal PFOA and PFOS levels in serum collected in 2^nd^ trimester from a subset of n = 88 mothers.

	PFOA		PFOS	
	Without GFR^2^	With GFR^3^	Without GFR	With GFR
	% change (95% CI)	% change (95% CI)	% change (95% CI)	% change (95% CI)
**GFR** (per 10 ml/min per 1.73 m2)^1^	-	2.6 (-7.6, 2.8)	-	**-8.6 (-15, -2.3)**
Maternal height (per 10 cm)	-12 (-24, 3.2)	-9.0 (-23, 7.3)	**-19 (-33, -0.7)**	-10 (-27, 10)
Maternal BMI (per kg/m^2^)	0.2 (-3.2, 3.6)	1.6 (-2.9, 6.3)	2.1 (-2.3, 6.6)	**7.4 (1.5, 14)**

^1^Mutually adjusted estimates, in addition to adjustment for gestational age at serum sampling.

^2^Analyses performed without adjustment for GFR.

^3^Analyses performed with adjustment for GFR.

#### OCs

In adjusted analyses, Swedish women had, on average, 43% higher levels of PCB 153 and 9% lower levels of *t*-NC than Norwegian women. All OC levels, except PCB 118 and *t*-NC, decreased from January 1986 through March 1988. Previous breastfeeding duration was negatively associated and maternal age positively associated with all OCs (Table 3). Pre-pregnancy BMI was negatively associated with PCB 153 and *t-*NC. Women who smoked at conception had on average 26% lower levels of PCB 118 and 9% higher levels of *β-*HCH, compared to non-smokers. In sensitivity analyses, we found a clear dose-response relationship between increasing number of cigarettes smoked both at conception and (p<0.001) and in early 2^nd^ trimester (p<0.001) and decreasing PCB 118 levels (Table 4). This finding was confirmed in a sub-sample analysis of the n = 88 women with available serum cotinine levels (p = 0.004). We also found some evidence of a positive linear relationship between cigarettes smoked at conception and in 2^nd^ trimester and *β*-HCH levels, but this could not be confirmed with cotinine levels (p = 0.428). Increased length of education was significantly associated with increased *p*,*p’-*DDE, *t-*NC, and *β-*HCH levels, and alcohol consumption during pregnancy was positively associated with serum PCB153, *t*-NC and HCB levels (Table 3).

## Discussion

In this descriptive study of n = 424 Scandinavian parous women, Swedish women had higher levels of PFOS, PFOA and PCB 153 levels compared to Norwegian ones. Our results indicate that several maternal factors, including potentially modifiable lifestyle factors, are independently associated with maternal serum EDC levels. We demonstrated that long previous breastfeeding duration was associated with lower PFAS and OC levels, and that long time since last breastfeeding period was associated with higher PFAS levels. Maternal smoking showed a consistently negative association with PCB 118 in a dose-dependent manner.

### Serum levels of PFASs and OCs; sampling date and geographic factors

Both maternal PFAS and OC levels found in our study were lower than levels among a group of Danish pregnant women sampled in 1988–1989 [24]. PCB levels were slightly lower than levels among women from the Netherlands (1990–1992) [25] and higher than levels among Swedish primiparous women enrolled in 1996–1999 [26]. Our study, conducted in 1986–1988, had only parous women that may have led to lower levels of PFASs and OCs due to elimination through the placenta and breast milk in earlier pregnancies.

The increase in PFAS levels over time (i.e. from January 1986 to March 1988) is consistent with other studies showing a rise in PFOA and PFOS levels from the 1960s to around 2000, and a subsequent decrease thereafter [27–29]. In contrast, almost all OC levels declined from January 1986, which is consistent with findings from longitudinal studies that observed a declining trend of most OCs after 1986 [30–32]. These changes compare with trends of historic production, restrictions, and use and bans on use of PFASs and OCs, leading to declining levels of these toxicants in air and biota [3].

Higher levels of PCBs among Swedish women seem reasonable and correspond to higher levels reported in fish from the Baltic Sea [33, 34]. Dietary intake is probably also the most important exposure source to PFASs [35], and particularly seafood in Scandinavian countries [36]. However, other sources of exposure such as drinking water and dust may contribute to variability in levels between the study sites, particularly in time periods with continued use and emissions [35, 37].

Despite declining serum levels of PFASs and OCs in most Western countries, biomonitoring programs still demonstrate high levels of both PFASs and OCs among certain populations and countries today [3]. So, this study may be of great importance to certain populations with still high environmental levels of PFASs and OCs, i.e. developing countries [14].

### Maternal socio-demographic and pregnancy related factors associated with PFAS levels

Our results emphasize that information on previous breastfeeding duration is important in the evaluation of PFAS levels in women. The negative linear association between breastfeeding duration and PFAS levels is also consistent with other studies [38, 39]. Due to the placenta barrier, elimination through breast milk is thought to be greater than the prenatal transfer to the fetus [40]. Several studies from post-ban periods (after year 2000) have found parity as an important predictor of PFAS levels [41]. The null association between parity and PFAS levels in the current study is in line with a study from the pre-ban period (1978–2001) when parity was not identified as a predictor of PFOS levels [42]. This suggests that the relative importance of parity as a predictor differs between pre- and post-ban periods. We found the same null association between parity and PFAS levels when we excluded breastfeeding duration from the multivariable models (data not shown). We also included time since last breastfeeding period in the analyses because the importance of parity likely depends on how recent the previous pregnancy was.

PFASs have the potential to bio-accumulate, and we hypothesize that non-pregnant periods without breastfeeding are important accumulation periods for women of fertile age. The positive association between PFAS levels and time since last breastfeeding period in the current study is consistent with a Norwegian study of pregnant women sampled in 2003 that also showed a rise in PFAS levels during pregnancy intervals [38]. Long intervals between two subsequent pregnancies have been associated with adverse perinatal outcomes [43], thus, it is important to consider that time period as a potential confounder in child health effect studies.

The lower levels of PFOS among women who smoked at conception were confirmed in additional analyses where increasing number of cigarettes smoked at conception and in early 2^nd^ trimester were associated with decreasing levels of PFOS. However, the non-linear relationship between serum cotinine and PFOS levels corresponds to other studies that found inconsistent associations with smoking overall, and no associations with smoking intensity [38, 44]. This suggests that the relationship between smoking and PFOS levels may be explained by different lifestyle patterns (e.g. diet) among smokers and non-smokers.

Only one other study has examined associations between GFR and PFASs among pregnant women [45], and like that study, we found an inverse association between GFR and PFOS (p = 0.002) in a sub-sample (n = 88). GFR describes the flow rate of filtered fluid through the kidney, and we postulate that higher GFR leads to more excretion of PFASs and thereby lower serum levels of PFASs. Because studies have indicated a possible association between GFR and infant birth weight [46], GFR might be considered a potential confounder in epidemiologic studies of PFASs and fetal growth. In fact, a recent study that examined the impact of GFR on associations between PFASs and birth weight, suggested that associations were largely attenuated due to confounding by GFR [47].

The weak negative association we observed between pre-pregnancy BMI and PFOS is in contrast to other studies that reported positive [7, 38, 45] or null associations [42, 48]. This finding, together with the negative association between maternal height and PFAS levels, is not completely understood, but may be due to proposed poorer plasma volume expansion, leading to higher PFAS levels, among smaller sized women [49]. BMI was not associated with maternal height in our study, but lack of adjustment for GFR might give biased results in the multivariate models. GFR is positively related to maternal height and weight. In a sensitivity analysis (n = 88), we estimated the associations between GFR, maternal height and BMI and PFAS levels. When we included GFR in the model, the negative association between maternal height and PFOS attenuated and the association between BMI and PFOS became positive (Table 5). This suggests that BMI and maternal height might be proxies for other variables, like GFR, and results in multivariate linear models should be interpreted with caution.

### Maternal socio-demographic and pregnancy related factors associated with OC levels

The strong inverse associations between breastfeeding duration and all OC levels in the current study are consistent with previous studies [50]. This is explained by excretion of OCs in the breast milk, resulting in lower maternal serum levels of OCs and exposure to breastfeeding children [51]. A recent review concluded that the benefits of breastfeeding far outweigh the potential disadvantages, but argues to plea for further global source-directed methods to reduce human exposure to OCs [52].

The positive association between maternal age and OC levels has been found in several other cross-sectional studies [33, 53]. Age indicates whether the subject lived during periods with higher exposure to OC levels. Based on longitudinal studies it has been shown that this association may be considered a cohort effect related to historical emission patterns, rather than age-dependent metabolism or bioaccumulation [32].

The inverse relationships between pre-pregnancy BMI and serum levels of PCB153 and *t-*NC levels may be explained by the dilution effects of OCs into adipose tissues [54]. However, obesity can also prolong the half-lives of OCs whereby positive associations can be seen as well [55]. Conversely, weight loss may lead to increased serum levels due to reduced storage capacity in the adipose tissues. Similarly, weight gain may lead to decreased serum levels through dilution of OCs into adipose tissue [56, 57]. The inverse association between BMI and OC levels as found in our study, is supported by other cross-sectional studies that have observed varying associations with BMI depending on the OC under study [58].

The lower levels of PCB 118 among smokers at conception in adjusted analyses, were further confirmed in additional analyses with smoking intensity both by number of cigarettes smoked at conception and at early 2^nd^ trimester, and by serum cotinine in 2^nd^ trimester (n = 88). These findings support the hypothesis that smoking cigarettes can enhance the metabolism of PCB118, leading to reduced levels in serum. It has been speculated that cigarette smoke can enhance the elimination rates of dioxins and dioxin-like PCBs, due to an induction of CYP-enzymes [59]. Reasons for higher levels of *β*-HCH among smokers are unclear, but several studies have found positive associations between OCs and smoking. This association may be attributed to either the use of these pesticides on tobacco plants until they were banned in the 1970s, or alternatively that cotinine influences the expression of CYP-enzymes that metabolize organochlorines [60].

The positive associations between education level and serum OC levels might be explained by different lifestyle patterns. More educated women with higher socio-economic status may have different dietary patterns, such as increased consumption of fish. The linear relationship between increased alcohol consumption and serum PCB153, *t-*NC and *β*-HCH levels might be attributed to changes in fatty acid composition [61]. However, the overall alcohol consumption was quite small, and other lifestyle factors might also have contributed to the differences in levels.

### Strengths and limitations

This study benefitted from a large sample size and detailed information about socio-demographic, lifestyle, pregnancy related factors, obstetric history and markers of pregnancy physiology, that have been considered in only a few epidemiological studies. The serum samples were taken in a narrow time frame in the early 2^nd^ trimester (from week 13–20) to ensure comparability. To ensure that the results were not biased by the enrichment of mothers with SGA offspring, we did stratum-weighted sensitivity analyses where weights were the inverse probability of selection [62]. We compared the results from the un-weighted and the weighted analyses, and found that they were not substantially different (S1 Table). Women expecting their first child were not included in the study, and this may have led to some selection bias, especially when considering excretion of EDCs through previous breastfeeding. We did not distinguish between *exclusive* and *partial* breastfeeding duration, and the lack of information about the exclusive breastfeeding duration may have led to residual confounding and spurious associations between PFASs and OCs and factors associated with breastfeeding duration. However, after birth we collected information about both *exclusive* and *partial* breastfeeding duration after the *current* pregnancy, and found that both exclusive and total (exclusive + partial) breastfeeding were highly correlated (r = 0.6). We also found total breastfeeding duration to be correlated between pregnancies, suggesting that potential bias is limited. Moreover, breastfeeding duration was recalled quite accurately 20 years after mothers gave birth in this study [63]. We selected our cohort from an original SGA study with a high risk for SGA birth group that included a high proportion of smokers. Although this may limit generalizability to other studies with fewer smokers, we were able to thoroughly study the relation between smoking status, smoking intensity and serum cotinine levels and serum PFAS and OC levels in an otherwise homogeneous population. To estimate GFR, we used an indirect method, based on a single-point measurement of serum creatinine, as opposed to inulin clearance, which is the gold standard method [64]. Renal function drastically changes during pregnancy, because of hyper filtration, systemic vasodilatation and plasma volume expansion, resulting in up to 60% increase in GFR compared to values obtained before pregnancy [64]. Hence, indirect methods may be biased. However, studies have shown that with increasing GFR, the differences between direct and indirect methods stayed the same, and that bias was limited [64].

## Conclusions

In summary, our study observed higher maternal serum PFOA, PFOS and PCB 153 levels among Swedish compared to Norwegian women. Several maternal factors including potentially modifiable lifestyle factors, markers of pregnancy physiology and important factors related to perinatal outcomes were associated with maternal serum EDC levels. Interestingly, we demonstrated that long time since last breastfeeding period was associated with higher PFAS levels and that maternal smoking showed a consistently negative association with PCB 118 in a dose-dependent matter. Results from this study are particularly relevant to populations with still high PFAS and OC levels, for instance in some developing countries. Moreover, we can use this knowledge about associated factors on new emerging EDCs with similar properties.

## Supporting Information

S1 Table Adjusted associations from multivariable linear regression models for sociodemographic and pregnancy-related variables and ln-transformed maternal PFAS and OC levels in serum from 2^nd^ trimester (n = 424): un-weighted and stratum-weighted analysis. (DOCX)
