# Supplementary material for: Factors Associated with Maternal Serum Levels of Perfluoroalkyl Substances and Organochlorines: A Descriptive Study of Parous Women in Norway and Sweden
Source: PLoS One. 2016 Nov 8;11(11):e0166127. doi: 10.1371/journal.pone.0166127 (PMC5100957; doi:10.1371/journal.pone.0166127)
Supplement: S1 Table — (DOCX) [file pone.0166127.s001.docx]

|  |  | **Ln (PFAS (ng/ml))** | | **Ln (OC (ng/g lipid))** | | | | | |
| --- | --- | --- | --- | --- | --- | --- | --- | --- | --- |
|  |  | **PFOA** | **PFOS** | **PCB118** | **PCB153** | ***p,p'*-DDE** | **HCB** | ***β*-HCH** | ***t*-NC** |
|  |  | % change (95% CI) | % change (95% CI) | % change (95% CI) | % change (95% CI) | % change (95% CI) | % change (95% CI) | % change (95% CI) | % change (95% CI) |
| Sample date (per 100 days) | A | **2.8 (0.7, 4.9)** | **5.0 (2.4, 7.7)** | -1.6 (-3.4, 0.2) | **-2.5 (-3.7, -1.3)** | **-7.2 (-9.7, -4.7)** | **-2.4 (-3.7, -1.0)** | **-4.4 (-5.9, -2.8)** | -0.5 (-2.2, 1.3) |
|  | B | **3.2 (1.0, 5.4)** | **5.1 (2.3, 7.9)** | -1.3 (-3.1, 0.6) | **-2.2 (-3.5, -0.9)** | **-6.4 (-9.0, -3.8)** | **-2.7 (-4.1, -1.3)** | **-4.7 (-6.3, -3.1)** | -0.5 (-2.3, 1.3) |
| Country of residence |  |  |  |  |  |  |  |  |  |
| Norway |  | *ref.* | *ref.* | *ref.* | *ref.* | *ref.* | *ref.* | *ref.* | *ref.* |
| Sweden | A | **39 (27, 53)** | **67 (49, 88)** | 8.0 (-0.7, 17) | **43 (35, 52)** | 2.0 (-11, 16) | -2.5 (-8.7, 4.2) | 7.4 (-0.5, 16) | **-9.4 (-16, -1.7)** |
|  | B | **34 (21, 47)** | **59 (41, 79)** | 7.6 (-1.4, 17) | **41 (33, 50)** | 0.6 (-11, 14) | -5.3 (-11, 1.1) | 3.8 (-3.8, 12) | **-10 (-17, -2.6)** |
| Maternal height (per 10 cm) | A | *-6.7 (-14, 0.3)* | *-8.3 (-16, 0.6)* | 5.6 (-1.1, 13) | 1.1 (-3.4, 5.9) | 10 (-0.6, 22) | 1.9 (-3.3, 7.3) | 0.1 (-5.7, 6.2) | 1.1 (-5.2, 7.7) |
|  | B | -4.3 (-11, 3.0) | -6.9 (-15, 2.1) | **7.6 (0.7, 15)** | 2.8 (-1.8, 7.5) | **13 (2.3, 24)** | 1.9 (-3.2, 7.1) | 1.7 (-4.0, 7.7) | 1.8 (-4.3, 8.4) |
| Maternal BMI (per kg/m^2^) | A | -1.4 (-3.0, 0.3) | **-2.3 (-4.2, -0.2)** | -0.5 (-1.9, 1.0) | **-2.6 (-3.6, -1.6)** | -1.1 (-3.3, 1.1) | -0.3 (-1.4, 0.9) | 1.0 (-0.3, 2.4) | **-2.0 (-3.4, -0.6)** |
|  | B | **-2.1 (-3.7, -0.4)** | **-3.0 (-5.0, -1.0)** | -0.5 (-1.9, 1.1) | **-2.2 (-3.2, -1.2)** | -1.2 (-3.4, 1.0) | -0.02 (-1.2, 1.1) | 1.6 (0.2, 2.9) | *-1.4 (-2.8, 0.01)* |
| Maternal age | A | -0.1 (-1.5, 1.3) | -0.1 (-1.9, 1.7) | **2.5 (1.2, 3.8)** | **2.9 (2.0, 3.8)** | **5.5 (3.5, 7.6)** | **2.6 (1.6, 3.7)** | **3.2 (2.0, 4.4)** | **3.5 (2.3, 4.8)** |
|  | B | 0.1 (-1.3, 1.6) | -0.2 (-2.0, 1.6) | **3.2 (1.9, 4.6)** | **3.2 (2.3, 4.1)** | **6.6 (4.5, 8.6)** | **2.9 (1.8, 3.9)** | **3.3 (2.1, 4.4)** | **3.8 (2.5, 5.1)** |
| Smoking at conception |  |  |  |  |  |  |  |  |  |
| No |  | *ref.* | *ref.* | *ref.* | *ref.* | *ref.* | *ref.* | *ref.* | *ref.* |
| Yes | A | -4.7 (-15, 5.0) | **-21 (-37, -7.4)** | **-26 (-38, -16)** | 0.9 (-5.3, 6.6) | 3.0 (-11, 15) | -4.4 (-12, 2.5) | **9.2 (1.9, 16)** | 7.0 (-1.1, 14) |
|  | B | -5.4 (-16, 4.5) | **-22 (-38, -7.6)** | **-24 (-35, -13)** | 1.7 (-4.5, 7.5) | 6.4 (-6.6, 18) | -5.2 (-13, 1.8) | *7.4 (-0.1, 14)* | **8.9 (0.9, 16)** |
| **Alcohol consumption** (5 groups from low to high)^4^ | A | 0.6 (-4.2, 5.5) | -0.4 (-6.2, 5.8) | **5.1 (0.8, 9.7)** | 3.5 (0.4, 6.6) | 3.3 (-3.2, 10) | 3.2 (-0.2, 6.8) | 2.5 (-1.3, 6.6) | 5.6 (1.3, 10) |
|  | B | -1.0 (-5.7, 3.8) | -1.7 (-7.4, 4.4) | **5.0 (0.5, 9.6)** | 3.8 (0.8, 6.9) | 4.3 (-2.2, 11) | 3.0 (-0.4, 6.4) | 2.6 (-1.2, 6.6) | 5.8 (1.5, 10) |
| **Education level** (5 groups from low to high)^3^ | A | 2.0 (-2.4, 6.6) | 3.0 (-2.5, 8.7) | 2.3 (-1.6, 6.3) | 1.6 (-1.1, 4.4) | **7.0 (0.9, 14)** | 1.4 (-1.7, 4.6) | 3.3 (-0.3, 7.0) | **4.8 (0.9, 8.8)** |
|  | B | 2.4 (-2.0, 7.0) | 2.4 (-3.1, 8.2) | 0.1 (-3.8, 4.2) | 0.4 (-2.3, 3.2) | 4.4 (-1.5, 11) | 0.3 (-2.7, 3.3) | 1.6 (-1.9, 5.2) | **3.8 (0.0, 7.8)** |
| **Parity** |  |  |  |  |  |  |  |  |  |
| 1 |  | *ref.* | *ref.* | *ref.* | *ref.* | *ref.* | *ref.* | *ref.* | *ref.* |
| 2 | A | -3.1 (-13, 7.9) | -6.7 (-19, 6.8) | 8.1 (-1.8, 19) | 7.2 (0.3, 15) | 7.6 (-7.1, 25) | 2.1 (-5.4, 10) | 1.2 (-7.2, 10) | 0.3 (-8.6, 10) |
|  | B | -0.0 (-10, 11) | -4.6 (-17, 9.1) | 8.0 (-2.0, 19) | 6.0 (-0.8, 13) | 3.9 (-10, 20) | 1.6 (-5.6, 9.4) | 2.6 (-5.7, 12) | 1.6 (-7.2, 11) |
| **Previous breastfeeding duration** (per month) | A | **-1.3 (-2.3, -0.2)** | **-1.6 (-2.8, -0.3)** | **-2.3 (-3.2, -1.4)** | **-2.1 (-2.7, -1.4)** | **-3.5 (-4.9, -2.2)** | **-1.9 (-2.6, -1.2)** | **-2.7 (-3.6, -1.9)** | **-1.0 (-1.9, -0.1)** |
|  | B | **-1.7 (-2.7, -0.6)** | **-1.8 (-3.1, -0.5)** | **-2.5 (-3.4, -1.6)** | **-2.1 (-2.8, -1.5)** | **-3.5 (-4.8, -2.1)** | **-2.0 (-2.7, -1.3)** | **-2.6 (-3.4, -1.8)** | **-1.3 (-2.1, -0.4)** |
| **Time since last breastfeeding period** (per year) | A | **4.8 (2.9, 6.8)** | **2.8 (0.5, 5.2)** | -0.5 (-2.1, 1.1) | -0.4 (-1.5, 0.7) | -0.6 (-3.0, 1.9) | -0.2 (-1.5, 1.0) | 0.2 (-1.2, 1.7) | -0.01 (-1.6, 1.6) |
|  | B | **4.8 (2.9, 6.8)** | *2.4 (-0.02, 4.8)* | -1.1 (-2.7, 0.6) | -0.7 (-1.9, 0.4) | -1.0 (-3.4, 1.5) | -0.1 (-1.4, 1.2) | 0.7 (-0.8, 2.2) | -0.2 (-1.8, 1.4) |

**S1 Table. Adjusted^1^ associations from multivariable linear regression models for sociodemographic and pregnancy-related variables and ln-transformed maternal PFAS (ng/ml) and OC (ng/g lipids) levels in serum from 2^nd^ trimester (n=424): un-weighted and stratum-weighted^2^ analysis.**

^1^Multivariable models were adjusted for all variables included in the table.

^2^Stratum-weighted: weights are the inverse probability of selection.

^3^Education level (ordinal): 1=<9 years, 2=9-11 years, 3=12 years, 4=higher education, non-university level, 5=higher education, university level.

^4^Alcohol consumption during pregnancy (ordinal): 0=never, 1=<once a month, 2=once a month, 3=2-3 times a month, 4=>once a week.

A: Un-weighted analysis, B: Stratum-weighted analysis
